# Dietitian‐led very low‐calorie diet for preoperative rehabilitation in patients with obesity awaiting non‐bariatric elective laparoscopic surgery: A retrospective study

**DOI:** 10.1002/ncp.70094

**Published:** 2026-02-03

**Authors:** Gerald Wei Shen Wong, Cathy Guo, Cameron M. French, Jack J. Bell, Lynda J. Ross

**Affiliations:** ^1^ School of Exercise and Nutrition Sciences, Queensland University of Technology Brisbane Queensland Australia; ^2^ Khoo Teck Puat Hospital, National Healthcare Group Singapore Singapore; ^3^ The Prince Charles Hospital Brisbane Queensland Australia; ^4^ Allied Health Research Collaborative, Metro North Hospital and Health Service Brisbane Queensland Australia

**Keywords:** laparoscopic surgery, obesity, preoperative weight loss, surgical time, VLCD

## Abstract

**Background:**

Obesity increases the risks and complexity of laparoscopic surgeries. Preoperative very low‐calorie diets (VLCDs) can demonstrate significant preoperative weight loss. However, the optimal VLCD duration remains unclear. Excessive loss of muscle mass associated with VLCDs may elevate surgical and postoperative risks. This study aimed to assess the impact of a dietitian‐led preoperative VLCD intervention on changes in weight, muscle, and fat mass and to examine their relationships with intervention duration and surgical time.

**Methods:**

A retrospective chart review of patients attending a dietitian‐led VLCD outpatient clinic for 1–8 weeks. Primary outcomes were changes in weight, muscle and fat mass, and their relationships with VLCD duration. Associations between preoperative fat mass and operative time for cholecystectomies and hernia repairs were explored using a general linear model.

**Results:**

One hundred fifty‐seven participants (97 female; mean body mass index, 39.2) achieved significant weight reduction (6.4 kg, *P* < 0.001). Muscle mass accounted for 28.5% of mean weight loss and fat mass for 68%, with an increased muscle to fat ratio (*P* < 0.001). VLCDs of >4 weeks showed greater median muscle mass loss (26.9% vs 8.8%). Operative time decreased by an estimated 0.61 min for every kilogram reduction in preoperative fat mass, after adjusting for surgical type (*P* < 0.001, adjusted *R* ^2^ = 0.262).

**Conclusions:**

A dietitian‐led preoperative VLCD intervention for 5–8 weeks can achieve clinically significant weight loss, primarily from fat mass. Exploratory analysis suggested lower preoperative fat mass may contribute to shorter surgical time, but further research is needed to control for other factors, such as complexity and surgeon experience.

## Graphical abstract

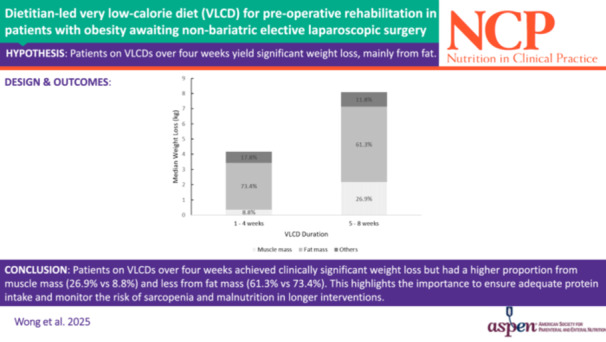

## INTRODUCTION

Obesity is a global public health issue that has reached epidemic proportions, with 1 billion adults predicted to live with obesity by 2030.^1^ Over the past two decades, Australia has experienced a steady rise in obesity prevalence, with approximately 5.8 million Australian adults living with obesity.^2^ The substantial growth in obesity has been linked to increases in other health conditions, such as gallstones and hernias, which require elective laparoscopic surgery.^3^, ^4^ However, excessive visceral fat and enlarged livers elevate both surgical risk and complexity in patients with obesity.^5^, ^6^ Excessive visceral fat and fat accumulation within the liver decrease its pliability, thereby increasing the technical complexity of liver manipulation to access the surgical site.^7^ Additionally, the fibrofatty liver has an increased risk of severe bleeding or potential fracture from retraction trauma, further complicating the surgical procedure.^8^ The increase in obesity‐related surgical complications has resulted in greater resource utilization and extended hospital stays, further intensifying the economic burden on healthcare systems.^9^

Preoperative weight loss programs are widely recognized for reducing obesity‐related surgical risks.^10^, ^11^ Very low‐calorie diets (VLCDs), also known as very low‐energy diets, currently demonstrate the most effective short‐term, nonsurgical, nonpharmacological weight loss for adults with obesity.^12^, ^13^ Although specific products and regimens may differ, VLCDs generally involve substituting one or more daily meals with foods or commercial products to provide <800 kcal/day.^13^ VLCDs are widely used in patients before laparoscopic bariatric surgery and have been shown to decrease surgical complication rates in bariatric surgery by promoting weight loss, reducing visceral fat, and shrinking liver volume.^14^, ^15^ The limited literature investigating VLCDs prior to elective non‐bariatric laparoscopic surgeries reported similar weight loss findings.^16^, ^17^, ^18^, ^19^ However, out of those studies, only one study with a small sample size (*n* = 25) examined muscle mass (MM) change.^16^ Sarcopenia resulting from malnutrition is a major concern associated with preoperative VLCDs, as it increases the risk of postoperative morbidity and mortality.^20^ Because of the limited evidence, the impact of preoperative VLCDs on MM in patients awaiting elective non‐bariatric laparoscopic surgery remains unclear. Preoperative VLCD durations in previous studies have varied from 2 to 12 weeks, depending on weight loss goals,^18^ liver volume reduction,^19^ and surgery waiting times.^17^ Thus, understanding the influence of preoperative VLCD durations on surgical risk factors is vital to designing an effective and safe intervention. Furthermore, research revealed that a large proportion of body fat mass (FM) loss occurred in visceral adipose tissue when using VLCDs for modest (3.9%–8.1%) weight loss.^15^ Decreased visceral adipose tissue improves laparoscopic access to the surgical site, shortening surgical time.^21^ Therefore, we hypothesize that a positive association exists between preoperative FM and surgical time of laparoscopic procedures.

The primary aims of this study were (1) to evaluate the impact of a dietitian‐led preoperative VLCD‐based model of care on changes in weight, MM, and FM in patients with obesity awaiting elective non‐bariatric laparoscopic surgery and (2) to examine the influence of VLCD duration on anthropometric changes. The secondary aims were to report key surgical outcomes of laparoscopic cholecystectomy and hernia repair procedures and investigate the influence of preoperative FM on these surgical times.

## MATERIALS AND METHODS

### Study design

This retrospective observational medical chart review targeted a convenience sample of patients with obesity awaiting elective non‐bariatric laparoscopic surgery, who underwent a dietitian‐led VLCD‐based model of care (hereafter referred to as “the VLCD model”) from July 27, 2018, to October 9, 2020, at a 630‐bed public tertiary hospital located in Brisbane, Queensland, Australia. The study was approved by The Prince Charles Hospital Human Research Ethics Committee (HREC/16/QPCH/178).

### Study sample

All patients referred to the VLCD model between July 1, 2018, and September 30, 2020, were identified from an established database and underwent eligibility screening by the researchers (GW, CG). Patients who were aged >18 years, had a body mass index (BMI; calculated as weight [kg] divided by height squared [m^2^]) ≥30, were referred by their surgeons, were awaiting non‐bariatric elective laparoscopic surgery at the hospital, attended at least one dietitian appointment, and verbally consented to participate in the VLCD model were eligible. The study excluded patients who were pregnant or preparing for/undergoing in vitro fertilization and patients with intellectual or mental impairment, liver failure, portal hypertension, acute cardiovascular disease, or type 1 diabetes. All participants were screened for malnutrition by dietitians using the Malnutrition Screening Tool,^22^ and those identified as at risk were excluded from the VLCD model.

### The VLCD model

In the general surgery outpatient clinic, the VLCD model was provided as standard practice for patients with obesity who were referred by their treating surgeons for preoperative weight loss prior to non‐bariatric elective laparoscopic surgery, with no run‐in period. All general surgeons at the hospital were informed of the study and VLCD model before its initiation. The study outline, inclusion criteria, and referral guidelines were discussed in detail. When a patient met the eligibility criteria, surgeons were prompted to refer the patient to the program, ensuring consistent recruitment across participating surgeons.

In the VLCD model, dietitians prescribed participants with an individualized VLCD for 1–8 weeks. A total of three dietitians were involved in delivering the VLCD model. Clinical judgment was used to individualize the length of the diet for each patient, guided by the published protocols produced by Optifast (Nestlé Health Science) to ensure consistency, safety, and evidence‐based practice across all dietitians.^23^, ^24^ All participants were on the intensive level (phase 1) of the Optifast weight loss program for rapid weight loss.^23^ Participants were instructed to consume three Optifast meal replacement shakes daily to restrict daily caloric intake to <800 kcal (~3400 kJ). Each shake contains 840–850 kJ (201–203 kcal), 20 g protein, 4.5–4.6 g fat, and 18.2–18.5 g carbohydrate. An additional shake was prescribed, if required, to meet each participant's protein requirements of at least 0.75 g of protein per kilogram of adjusted body weight per day to preserve MM.^24^

All Optifast shakes were provided to participants at no cost to promote compliance and incentivize weight loss. All costs for the VLCD program, including the Optifast shakes, were covered under the general surgery budget and the allocated funding for dietetic care. As the clinic is part of a public hospital, there was no charge to patients for dietitian visits; time allocated from the existing funded caseload was used to support the VLCD model. The cost of the Optifast shakes was specifically funded by the general surgeons through their allocated departmental budget. No blinding was implemented in this study. Surgeons were updated every 4 weeks on the progress of their patients in the VLCD program, including diet adherence and duration, to facilitate ongoing clinical management.

Participants attended two to three dietitian appointments during their VLCDs, including a baseline appointment, an end‐of‐program appointment before surgery, and a midpoint appointment if the VLCD duration exceeded 4 weeks. During the appointments, dietitians evaluated patients for eligibility to undergo VLCD and provided guidance on symptom management, adherence promotion, and the transition to a healthy diet after surgery. Although diet adherence was not formally assessed, it was inferred from participants’ self‐reported diet history, tolerance of VLCD, dietitian clinic attendance, and changes in anthropometric measurements. To support adherence, meal replacements were provided at each scheduled appointment. Patients were monitored for adverse effects, including tolerance to VLCD and symptoms such as headache, dizziness, and constipation, during scheduled dietitian appointments. Those unable to tolerate the VLCD were removed from the study. Although there were no routine medical reviews, all participants were medically cleared by their treating surgeons before starting the VLCD program. If any medication adjustments were required (eg, insulin), the patient's general practitioner was also contacted to provide input and clearance. Eligibility for surgery was determined by the surgeon's clinical judgment, including whether the patient had achieved sufficient weight loss to safely proceed with the operation. As this clinic and program were novel, there was no previously established standard for dietitian intervention in general surgery patients.

### Data collection and outcomes

All data, including participant characteristic data, characteristics of the VLCD model, anthropometric measurements, and surgical outcomes, were collected retrospectively from electronic and paper medical charts. Participant characteristic data encompassed age, sex, and type of surgery, whereas characteristics of the VLCD model comprised VLCD duration, dietitian clinic attendance rate, and duration on the surgical waitlist. Weight, MM, and FM were measured using a bioelectrical impedance analysis scale (BC‐420SMA, Tanita), whereas waist circumference was measured by dietitians using a tape measure. Clinically significant weight loss was defined as ≥5% of the baseline weight, as it has been shown in previous studies to decrease overall postoperative complications by 13%–18% and reduce liver volume by up to 23%.^25^, ^26^ Physical activity was not measured in this study, as no equations requiring a physical activity factor were used. Surgical outcomes included surgical time, length of hospital stay, postoperative complications, and hospital readmissions.

### Statistical analysis

Observational data were analyzed using descriptive statistics. Mean (SD) displayed normally distributed data, whereas median (range) presented data displaying a skewed distribution. The normality of variables was analyzed using histograms. The primary outcomes of this study were changes in body composition, including MM and FM, and assessment of sarcopenia. Comparisons between baseline and post‐VLCD anthropometric variables were analyzed using a paired‐samples *t* test or Wilcoxon signed rank test as appropriate. An independent‐samples *t* test or Mann‐Whitney *U* test was performed as appropriate to assess whether the changes in anthropometric data before and after the VLCD model varied based on VLCD duration. Anthropometric variables were analyzed using an intention‐to‐treat approach, where the Initial Observation Carried Backward method and the Last Observation Carried Forward method were used for analytical purposes for participants who did not attend the baseline or end‐of‐program appointment.

Surgical outcomes, including surgical time, length of hospital stay, postoperative complications, and hospital readmissions, were examined for potential associations with preoperative FM. However, the study was underpowered for all outcomes except surgical time. Therefore, surgical time was chosen as an exploratory outcome to evaluate its association with preoperative FM. A general linear model (GLM) was developed with guidance from an independent biostatistician to explore this potential association for laparoscopic cholecystectomies and umbilical, ventral, and inguinal hernia repairs. This analysis is exploratory because FM is only one of many factors that may influence surgical time which cannot be accounted for in this study. Nonetheless, the relationship between FM and surgical time was of interest to the research team FM. Other elective surgeries were excluded from the analysis because their diverse nature could introduce variability which compromises the generalizability of the findings. This also transformed the data distribution for surgical time from skewed to normal, a prerequisite for GLMs. A bidirectional stepwise approach was used to select variables for the GLMs. First, several primary GLMs independently assessed the influence of preoperative FM and various potential confounders (age, sex, and surgery type) on surgical time. Subsequently, all independent variables that exhibited a *P* value of <0.2 in the primary models were incorporated into a secondary GLM to examine their influence on surgical time while controlling for other independent variables.^27^ Nonsignificant predictors were removed if they were not identified as confounders using the 10% rule of confounding.^28^ Statistical analyses were performed using SPSS, version 29.0 (IBM Corp). Data were considered statistically significant when *P* values < 0.05.

## RESULTS

**Figure 1:**
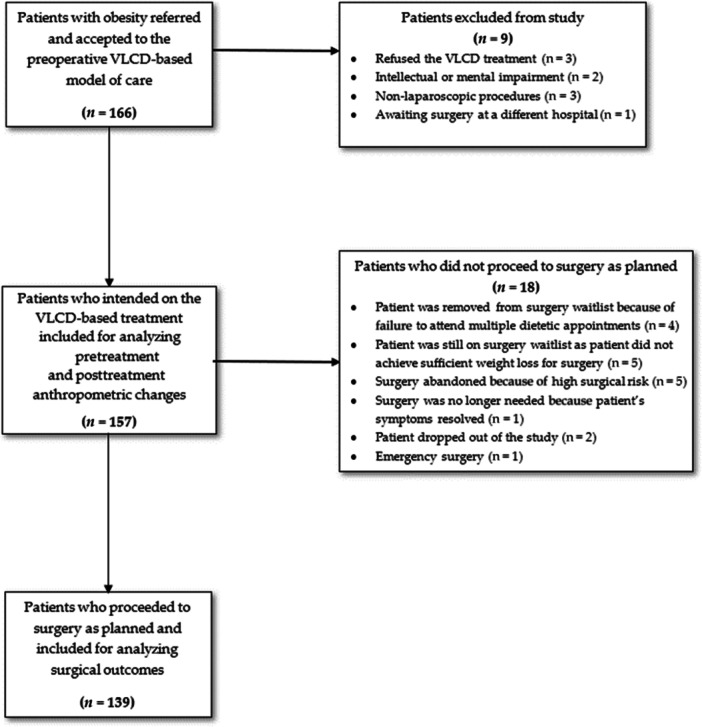
Flowchart of participant recruitment and analysis. Abbreviation: VLCD, very low‐calorie diet.

A total of 166 patients were referred to the VLCD model from July 1, 2018, to September 30, 2020. Out of them, 157 patients met the eligibility criteria, underwent the VLCD model, and were analyzed for anthropometric changes. Among these 157 patients, 139 (88.5%) patients proceeded to surgery as planned and were analyzed for surgical outcomes. No patients were removed from the study because of adverse effects or intolerance to VLCD. A flow diagram depicting the recruitment process and the progression of patients to surgery is shown in Figure 1.

### Characteristics of participants and the VLCD model

Table 1 presents the participants’ characteristic data, the characteristics of the VLCD model, and the baseline anthropometric data of participants. The age of participants ranged from 18 to 79 years, and 62% were female. Laparoscopic cholecystectomy was the most common surgical intent, accounting for 48% (*n* = 76) of all planned procedures. The most common VLCD duration was 7–8 weeks (40%, *n* = 63). Participants attended 94% (*n* = 377) of the total 400 dietitian appointments offered. The median duration between a participant's last attended dietitian appointment and surgery was 6 days. The mean (SD) BMI for participants at baseline was 39.2 (6.5), with 35% (*n* = 55) of participants with a BMI of ≥40 (class III obesity). The study included three participants who exhibited overweight status at baseline (initial dietitian consultation), as they had a BMI of ≥30 during their referral. Of the participants, 79.4% required four Optifast shakes because their protein requirements exceeded the approximate 58 g/day provided in three shakes.

**Table 1:** Characteristic data of 157 patients with obesity who had planned elective non‐bariatric laparoscopic surgery and underwent the VLCD model.

	Data
Patient characteristics	
Age, mean (SD), years	51.4 (13.5)
Female sex, *n* (%)	97 (62)
Surgery type, *n* (%)	
Cholecystectomy	76 (48)
Umbilical hernia repair	30 (19)
Ventral hernia repair	15 (10)
Inguinal hernia repair	19 (12)
Other^a^	17 (11)
Characteristics of the VLCD model	
VLCD duration, *n* (%)	
1–2 weeks	20 (13)
3–4 weeks	59 (38)
5–6 weeks	15 (10)
7–8 weeks	63 (40)
Dietitian clinic attendance	
Total number of appointments attended (% of total appointments offered, *n* = 400)	377 (94)
Duration on the surgical waitlist,^b^, ^c^ median (range) (*n* = 139), days	6 (0–701)
Baseline BMI, *n* (%)	
Overweight (25–29.9)	3 (2)
Obese class I (30–34.9)	45 (29)
Obese class II (35–39.9)	54 (34)
Obese class III (≥40)	55 (35)

*Note*: Data may not add up to 100% as a result of rounding.

Abbreviations: BMI, body mass index; VLCD, very low‐calorie diet.

^a^ Hiatus hernia repair + fundoplication (*n* = 3); high anterior resection (*n* = 2); right hemicolectomy (*n* = 2); appendicectomy + partial cecectomy + umbilical hernia repair (*n* = 1); low anterior resection + partial cecectomy (*n* = 1); cholecystectomy + umbilical hernia repair (*n* = 1); cholecystectomy + liver resection (*n* = 1); inguinal + umbilical hernia repair (*n* = 1); hiatus hernia repair (*n* = 1); right hemicolectomy (*n* = 1); bilateral femoral hernia repair (*n* = 1); appendectomy (*n* = 1); and resection of small bowel lesion (*n* = 1).

^b^ Participants who proceeded to elective non‐bariatric laparoscopic surgery.

^c^ Duration was defined as the time from the participant's last attended dietitian appointment to surgery.

### Anthropometric outcomes

Table 2 shows the anthropometric parameters before and after the VLCD model. Participants achieved a mean weight loss of 6.3 kg (5.6%) (*P* < 0.001). Over half of all participants (54%, *n* = 85/157) achieved clinically significant weight loss (≥5% baseline weight), with one participant gaining 1.7 kg. Among the 55 participants with class III obesity (BMI ≥ 40) at baseline, 22% (*n* = 12) lost sufficient weight to achieve a BMI of <40. Despite a statistically significant reduction in MM (1.8 kg, *P* < 0.001), the reduction only accounted for 28.5% of total weight loss. Contrastingly, the mean (SD) reduction in FM was 4.3 (3.7) kg (*P* < 0.001) and accounted for 68% of weight loss, leading to a mean (SD) increase in muscle to fat ratio of 0.2 (0.3) (*P* < 0.001).

**Table 2:** Anthropometric data of 157 participants with obesity who had planned elective non‐bariatric laparoscopic surgery, before and after VLCD intervention.

Outcome	Before VLCD, mean (SD)	After VLCD, mean (SD)	Change, mean (SD)	*P* value^a^
Weight, kg	111.7 (20.9)	105.4 (19.9)	−6.3 (4.3)	**<0.001**
BMI	39.2 (6.5)	37.0 (6.3)	−2.2 (1.5)	**<0.001**
Muscle mass (*n* = 155),^b^, ^c^ kg	61.0 (12.5)	59.2 (11.4)	−1.8 (3.4)	**<0.001**
Fat mass (*n* = 153),^d^, ^e^ kg	46.6 (15.1)	42.3 (14.7)	−4.3 (3.7)	**<0.001**
Muscle to fat ratio (*n* = 153)^f^	1.4 (0.5)	1.6 (0.6)	0.2 (0.3)	**<0.001**
Waist circumference (*n* = 155),^g^ cm	117.9 (13.3)	112.6 (12.3)	−5.3 (4.0)	**<0.001**
Female, cm	115.0 (12.6)	110.0 (11.8)	−4.8 (3.5)	**<0.001**
Male, cm	122.6 (13.1)	116.3 (12.1)	−6.4 (4.2)	**<0.001**

*Note*: Statistically significant *P* values < 0.05 are highlighted in bold.

Abbreviations: BMI, body mass index; VLCD, very low‐calorie diet.

^a^ Paired‐samples *t* test.

^b^ Missing data (*n* = 2): Participant could not get on the bioelectrical impedance analysis (BIA) machine (*n* = 1); measurement was not recorded in the medical chart because of clinician error (*n* = 1).

^c^ Muscle mass was defined as the bone‐free lean mass tissue.

^d^ Missing data (*n* = 4): Participant could not get on the BIA machine (*n* = 1); measurement was not recorded in the medical chart because of clinician error (*n* = 3).

^e^ Fat mass was defined as the total weight of fat mass in the body.

^f^ Muscle to fat ratio was characterized as the total weight of muscle (kg)/the total weight of body fat (kg).

^g^ Missing data (*n* = 2): Measurement was not recorded in the medical chart because of clinician error (*n* = 2).

### The VLCD duration and anthropometric outcomes

**Figure 2:**
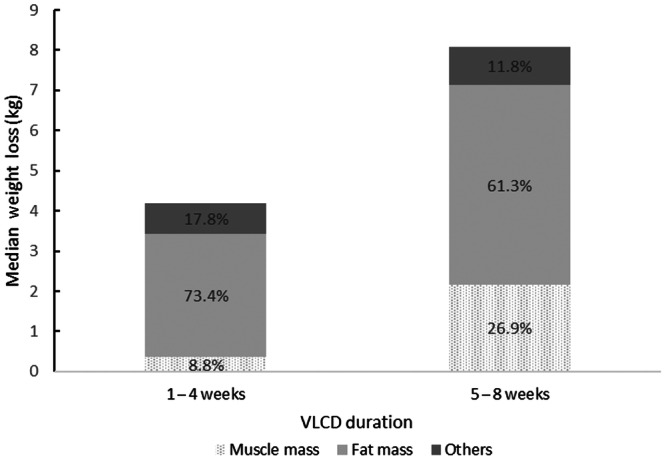
Proportion of muscle and fat in total weight loss for patients undergoing 1–4 weeks and 5–8 weeks of very low‐calorie diet (VLCD).

Table 3 shows pre‐VLCD and post‐VLCD anthropometric changes for participants who followed the VLCD for 1–4 weeks and those who followed for 5–8 weeks. Significantly greater reductions in all anthropometric parameters were observed in VLCDs lasting >4 weeks. Participants following VLCDs for >4 weeks achieved clinically significant mean weight loss compared with those with shorter VLCDs (7.2% vs 4.1%, respectively). Among 85 participants who achieved clinically significant weight loss, 59 (69.4%) had VLCDs of >4 weeks. However, they also had a higher proportion of total weight loss from MM (26.9% vs 8.8%) and a lower proportion from FM (61.3% vs 73.4%). Figure 2 illustrates the proportions of muscle and fat in total weight loss for patients undergoing 1–4 weeks and 5–8 weeks of VLCD.

**Table 3:** Comparison of the anthropometric changes of 157 participants with obesity who had planned elective non‐bariatric laparoscopic surgery and prescribed a VLCD for 1–4 or 5–8 weeks.

Outcome	1–4 Weeks (*n* = 79), median (range)	5–8 Weeks (*n* = 78), median (range)	*P* value^a^
Weight loss, %	4.1 (−1.2 to 15.7)	7.2 (0–17.5)	**<0.001**
Weight loss, kg	4.2 (−1.7 to 18)	8.1 (0–24.3)	**<0.001**
Reduction in muscle mass, kg^b^	0.7 (−15.1 to 10.2)	2.3 (−2.9 to 17.5)^c^	**<0.001**
Reduction in fat mass, kg^d^	3.2 (−2.6 to 19.8)	5.2 (−7.7 to 13.9)^e^	**<0.001**
Muscle to fat mass ratio	0.2 (−0.4 to 2.2)	0.1 (−1.1 to 0.5)	0.829
Reduction in waist circumference, cm	3.0 (0–16)^f^	7.0 (0–21)^f^	**<0.001**

*Note*: Statistically significant *P* values < 0.05 are highlighted in bold.

^a^ Mann‐Whitney *U* test.

^b^ Muscle mass was defined as the bone‐free lean mass tissue.

^c^ Missing data (*n* = 2): Participant could not get on the bioelectrical impedance analysis (BIA) machine (*n* = 1); measurement was not recorded in the medical chart because of clinician error (*n* = 1).

^d^ Fat mass was defined as the total weight of fat mass in the body.

^e^ Missing data (*n* = 4): Participant could not get on the BIA machine (*n* = 1); measurement was not recorded in the medical chart because of clinician error (*n* = 3).

^f^ Missing data (*n* = 1): Measurement was not recorded in the medical chart because of clinician error.

### Surgical outcomes

Table 4 shows the secondary outcomes of interest, surgical outcomes of participants who underwent surgery types included in this study. The median surgical time across all surgery types was 73 min. Overall, the median length of hospital stay was 1 day. Two participants with outlying lengths of stay (11 and 13 days) had protracted preoperative stays for bowel preparation. Two patients experienced mild to moderate postoperative complications (*n* = 2/139, 1.4%). The incident of hospital readmission in 30 days occurred in nine participants (6.5%).

**Table 4:** Surgical outcomes of 139 participants with obesity who had elective non‐bariatric laparoscopic surgery after the VLCD model.

Surgical outcome	Data
Surgical time, median (range), min^a^	
All surgeries	73 (21–372)
Cholecystectomy	72 (39–146)
Umbilical hernia repair	61 (21–129)
Ventral hernia repair	80 (49–163)
Inguinal hernia repair	85 (56–148)
Other^b^	107 (68–372)^c^
Length of hospital stay, median (range), days	
All surgeries	1 (0–13)
Cholecystectomy	1 (0–11)^d^
Umbilical hernia repair	1 (0–3)
Ventral hernia repair	2 (1–4)
Inguinal hernia repair	1 (1–2)
Other^b^	2 (1–13)^e^
Postoperative complication,^f^ *n*/*N* (%)	
All surgeries	2/139 (1.4)
Cholecystectomy	0/65 (0.0)
Umbilical hernia repair	0/28 (0.0)
Ventral hernia repair	0/14 (0.0)
Inguinal hernia repair	1/18 (5.6)^g^
Other^b^	1/14 (6.7)^h^
Hospital readmission within 30 days,^i^ *n*/*N* (%)	
All surgeries	9/139 (6.5)^j^
Cholecystectomy	4/65 (6.2)
Umbilical hernia repair	1/28 (3.6)
Ventral hernia repair	1/14 (7.1)
Inguinal hernia repair	1/18 (5.6)
Other^b^	2/14 (14.3)

^a^ Surgical time was defined as the duration between the administration of anesthesia and the completion of the surgical procedure.

^b^ Hiatus hernia repair + fundoplication (*n* = 3); high anterior resection (*n* = 2); right hemicolectomy (*n* = 2); appendicectomy + partial cecectomy + umbilical hernia repair (*n* = 1); low anterior resection + partial cecectomy (*n* = 1); cholecystectomy + umbilical hernia repair (*n* = 1); cholecystectomy + liver resection (*n* = 1); inguinal + umbilical hernia repair (*n* = 1); hiatus hernia repair (*n* = 1); right hemicolectomy (*n* = 1); bilateral femoral hernia repair (*n* = 1); appendectomy (*n* = 1); and resection of small bowel lesion (*n* = 1).

^c^ A participant with a surgical time of 372 min was an extreme outlier who underwent a combined procedure of low anterior resection and cecectomy.

^d^ A participant with a length of stay of 11 days was an extreme outlier who underwent preoperative preparation, such as bowel preparation.

^e^ A participant with a length of stay of 13 days was an extreme outlier who underwent preoperative preparation, such as bowel preparation.

^f^ Complications were defined as per the Clavien‐Dindo classification.

^g^ Postoperative wound infection (grade I complication).

^h^ Prolonged diarrhea (grade II complication). Participant underwent right hemicolectomy.

^i^ Hospital readmissions were defined as instances in which participants were readmitted to the hospital within 30 days after their surgery because of surgery‐related issues.

^j^ Abdominal pain (*n* = 4), postoperative wound infection (*n* = 2), shoulder pain (*n* = 1), back pain (*n* = 1).

### Preoperative FM and surgical time

In the primary GLMs, preoperative FM (*P* = 0.002), surgery type (*P* < 0.001), and sex (*P* = 0.017) had *P* values < 0.2 (Table S1). After including these three independent variables in the secondary GLM, both preoperative FM (*P* < 0.001) and surgery type (*P* < 0.001) remained independently associated with surgical time, whereas sex became insignificant (Table S2). Table 5 illustrates the results of the final GLM, which explained 26.2% of the variance in the surgical time. Notably, preoperative FM was found to be positively associated with the surgical time independent of surgery type. In this exploratory analysis, surgical time is predicted to decrease by 0.61 min for every kilogram decrease in preoperative FM, after adjusting for surgical type, but unable to be adjusted for other confounding factors such as surgeon experience.

**Table 5:** General linear model analysis for surgical time of 125 participants with obesity after very low‐calorie diet intervention.

	Adjusted *R* ^2^	Crude estimate	95% CI	*P* value
General linear model	0.262			**<0.001**
Intercept		52.72		**<0.001**
Preoperative fat mass		0.61	0.30–0.91	**<0.001**
Surgery type				**<0.001**
Cholecystectomy^a^		0		
Umbilical hernia repair		−21.23	−31.64 to −10.83	**<0.001**
Ventral hernia repair		5.30	−8.04 to 18.64	0.433
Inguinal hernia repair		18.68	6.01–31.35	**0.004**

*Note*: Statistically significant *P* values < 0.05 are highlighted in bold. Dependent variable: surgical time.

^a^ Reference category.

## DISCUSSION

This retrospective cohort study demonstrated that patients with obesity awaiting non‐bariatric elective laparoscopic surgery successfully achieved clinically significant preoperative weight loss with minimal MM reduction through a preoperative dietitian‐led VLCD‐based model of care. The study also found that longer VLCDs (5–8 weeks) resulted in significantly greater reductions in anthropometric measurements than shorter VLCDs (1–4 weeks).

Participants in the present study achieved a statistically and clinically significant mean weight reduction of 5.6% after undergoing the VLCD model. This resulted in 88.1% of participants achieving adequate weight loss to become eligible for surgery. Prior studies using VLCD interventions before non‐bariatric elective surgery have documented comparable preoperative weight loss ranging from 4% to 15%.^16^, ^19^, ^29^, ^30^, ^31^ Notably, in the current study, 22% of participants with class III obesity (BMI ≥ 40) had theoretically improved their American Society of Anesthesiologists (ASA) physical status classification by achieving a BMI <40.^32^ Prior research suggests this decreases their risk of prolonged hospital stays, postoperative complications, extended surgical time, and mortality.^33^

In the present study, less than a third of weight loss was attributed to MM, with most (68%) coming from FM. This aligns with findings from other studies investigating the impact of VLCDs on body composition, which reported minimal reductions in MM with 69%–85% of weight loss coming from FM.^16^, ^34^, ^35^, ^36^, ^37^ The reduction in MM was expected as a consequence of the significant weight loss. Furthermore, participants in the current study increased their muscle to fat ratio, which is often associated with a reduction in metabolic risks.^38^ Nonetheless, there are several limitations associated with using bioelectrical impedance analysis to assess body composition in individuals with obesity. The relatively high amount of total body water and extracellular water in individuals with obesity can result in underestimated FM and overestimated MM.^39^ Although more sophisticated methods, such as dual‐energy x‐ray absorptiometry, provide greater accuracy and precision in body composition analysis, bioelectrical impedance analysis was chosen for this population because of its ease of use and lower cost, making it more feasible for routine clinical practice.^40^

Excessive MM loss resulting from inadequate dietary protein intake can increase postoperative morbidity and mortality risks.^41^ Hence, it was crucial to ensure that the individualized protein requirements of participants in the present study were met to minimize MM reduction. The need for an additional Optifast shake to meet the protein requirements for 79.4% of participants in this study highlights the importance of individualized guidance from a dietitian. Interestingly, another study that implemented an 8‐week VLCD intervention tailored to individualized protein requirements prior to non‐bariatric elective abdominal surgery also observed minimal loss of MM with significant weight reduction.^16^ The additional Optifast shake to meet the individualized protein requirements of participants in both this study and the aforementioned study,^16^ resulting in a diet that exceeded the caloric restrictions associated with VLCDs, did not seem to compromise the weight loss of participants in either study. To monitor the risk of sarcopenia in preoperative patients undergoing VLCD, future studies may consider using diagnostic criteria established by international working groups, such as the European Working Group on Sarcopenia in Older People (EWGSOP2) and the Asian Working Group on Sarcopenia (AWGS).^42^, ^43^ These criteria classify sarcopenia based on specific cutoffs for MM, muscle strength, and physical performance. Additionally, malnutrition risk may be assessed using functional capacity, subcutaneous fat, and muscle wasting assessments within the Subjective Global Assessment tool.^44^ Other criteria within the tool, such as weight change, dietary intake, and gastrointestinal symptoms, may not be applicable as they are influenced by VLCD.

The largest proportion of participants who achieved clinically significant weight loss followed a VLCD for >4 weeks. Although numerous studies have reported similar clinically significant weight loss after an 8‐week VLCD program prior to non‐bariatric elective surgery^16^, ^31^, ^45^ and participants with a VLCD duration of <4 weeks in two other studies achieved a mean weight loss of <5%,^35^, ^46^ the VLCD duration depends on the purpose of the intervention. Besides weight loss, a reduction in hepatic volume and steatosis has been associated with a short (1–2 weeks) preoperative VLCD, which decreased intraoperative blood loss during the liver resection procedure.^47^ Liver shrinkage could also benefit patients undergoing laparoscopic cholecystectomy, as it eases the visualization and dissection of Calot's triangle.^48^

A reduction of abdominal adiposity and liver volume may enhance surgical access and dissection, reduce technical complexity, and ultimately, shorten operative time.^8^, ^49^ A 2‐week preoperative VLCD has demonstrated a significant reduction in liver volume on ultrasound and computed tomography scans in patients with obesity.^8^, ^50^ Accordingly, the Society of American Gastrointestinal and Endoscopic Surgeons (SAGES) recommends the use of preoperative VLCDs to reduce liver volume and improve access for laparoscopic bariatric procedures.^51^ This could be one possible explanation why lower preoperative FM was an independently significant predictor of shorter surgical time of laparoscopic cholecystectomies and hernia repairs in this study's exploratory analysis. However, this study did not account for potential confounding variables such as surgeon experience, involvement of trainees, complexity of the surgery, or the presence of assistants. These factors were not collected or included in the analysis, and therefore, the observed association should be interpreted cautiously and considered hypothesis‐generating rather than definitive.

The benefits of preoperative VLCDs may extend beyond bariatric surgeries. Reductions in both surgical time and perceived surgical complexity by surgeons after a VLCD program were observed in both bariatric and non‐bariatric procedures.^19^, ^31^, ^52^ For instance, Burnard et al demonstrated a 20% reduction (6 min; *P* = 0.009) in surgical time when a 2‐week VLCD program was implemented prior to laparoscopic cholecystectomy in patients with obesity.^19^ Potential reduction in surgical time through VLCDs may increase surgical caseloads and training opportunities for surgical trainees and could lead to in substantial hospital cost savings, given that the average cost of running an operating theater cost in Australia is approximately $2500 per hour.^53^ Nevertheless, a comprehensive cost‐benefit analysis is required in future studies to compare the expenses and savings associated with VLCDs.

Beyond these potential benefits, the VLCD model demonstrated good adherence, as evidenced by the large proportion of participants achieving adequate weight loss to become eligible for surgery and a high dietitian clinic attendance rate of 94%. This figure falls in the upper range of adherence for preoperative VLCD programs lasting 2–12 weeks, which ranges from 30% to 97%.^16^, ^29^, ^30^, ^31^, ^35^ The model also demonstrated that participants in the present study experienced a shorter median surgical waitlist duration of 6 days compared with the 205 days observed for category 3 general surgery at Queensland Health public hospitals during the study period.^54^ Another notable observation in the current study is that only two participants who followed the VLCD model experienced mild to moderate postoperative complications. Similarly, a previous randomized controlled trial on patients with obesity undergoing non‐bariatric elective abdominal surgery at the same hospital as in the present study also reported that no participants experienced severe postoperative complications after a preoperative VLCD program.^16^

Despite the positive findings, this study presents several limitations. Its small sample size and single‐center design limit its generalizability to a broader population and may introduce type II errors. Although it has a small sample size, this study is the largest study to date to assess the effects of preoperative VLCDs on non‐bariatric patients with obesity. The study is further limited by the absence of an appropriate control group and by not controlling for the comorbidities or physical activity levels of participants. Additionally, the retrospective clinical audit methodology is a potential source of missing data and/or bias. Moreover, the study design precluded evaluation of sarcopenia on longer‐term patient‐centered outcomes such as functional status, quality of life, or cost‐effectiveness. We are unable to conclude the longer‐term effectiveness of the intervention on patient or healthcare outcomes. Although no participants were removed from the study as a result of adverse effects or intolerance to VLCD, we acknowledge that gallstones and complications associated with rapid weight loss should be monitored. Gallstones and electrolyte imbalances were not specifically tracked in this study, so future research and clinical practice may consider incorporating these assessments. Differences in visceral and subcutaneous fat distribution between men and women were not measured in this study. This could be a possible reason why sex dropped out of the GLM. Although beyond the scope of the current project, assessing fat distribution may provide valuable insights into body composition changes and surgical outcomes. Future studies could consider incorporating these measurements to better understand sex‐specific responses to preoperative VLCD interventions.

## CONCLUSION

The present study demonstrated that patients with obesity awaiting non‐bariatric elective laparoscopic surgery could achieve significant preoperative weight reduction, primarily from FM, through a dietitian‐led VLCD‐based model of care. Furthermore, exploratory analyses identified lower preoperative FM may contribute to shorter surgical time for laparoscopic cholecystectomies and hernia repairs. Considering the good adherence and minimal postoperative complications reported in the present and previous studies, a preoperative dietitian‐led VLCD‐based model of care may be beneficial for appropriate patients with obesity undergoing non‐bariatric elective laparoscopic surgery. Given the projected rise in obesity and demand for non‐bariatric elective laparoscopic surgeries, prospective randomized controlled trials with a large multicenter sample are warranted to assess the efficacy of the VLCD model in improving surgical outcomes and its cost‐effectiveness.

## AUTHOR CONTRIBUTIONS

Cameron M. French contributed to the conception of the research. All authors contributed to the design of the research. Cameron M. French contributed to the acquisition of data. Gerald Wei Shen Wong and Cathy Guo equally contributed to acquisition, analysis, interpretation of data, and drafted the manuscript. All authors critically revised the manuscript, agree to be fully accountable for ensuring the integrity and accuracy of the work, and read and approved the final manuscript.

## CONFLICT OF INTEREST STATEMENT

None declared.

## Supporting information

Supplementary File for review.
