# Supplementary material for: Dietitian‐led very low‐calorie diet for preoperative rehabilitation in patients with obesity awaiting non‐bariatric elective laparoscopic surgery: A retrospective study
Source: Nutr Clin Pract. 2026 Feb 3;41(3):892–904. doi: 10.1002/ncp.70094 (PMC13193523; doi:10.1002/ncp.70094)
Supplement: Supplementary file 1 — Supplementary File for review. [file NCP-41-892-s001.pdf]

Table S1. Primary general linear model analyses of potential variables affecting the surgical time of 125 patients with obesity who underwent laparoscopic cholecystectomies or umbilical, ventral, or inguinal hernia repairs after treatment with the VLCD model

	**Adjusted R^2**	**Crude Estimate**	**95% CI**	**p-value**
Primary general linear models				
Age	0.002	-0.20	-0.54 to 0.15	0.256
Gender	0.038			**0.017**
Male [a]		0		
Female		11.53	2.14 to 20.91	**0.017**
Surgery Type	0.137			**< 0.001**
Laparoscopic cholecystectomy [a]		0		
Umbilical hernia repair		-20.25	-31.29 to -9.22	**< 0.001**
Ventral hernia repair		4.15	-9.83 to 18.14	0.558
Inguinal hernia repair		12.41	-0.60 to 25.42	0.061
Post-VLCD fat mass	0.066	0.52	0.19 to 0.86	**0.002**

Statistically significant *p*-values < 0.05 are highlighted in bold.

Abbreviation: CI: Confidence interval.

Dependent variable: Surgical time.

[a] Reference category.

Table S2. Secondary general linear model analysis of potential variables affecting the surgical time of 125 patients with obesity who underwent laparoscopic cholecystectomies or umbilical, ventral, or inguinal hernia repairs after treatment with the VLCD model

	**Adjusted R^2**	**Crude Estimate**	**95% CI**	**p-value**
Secondary general linear model	0.259			**< 0.001**
Intercept		49.77		**< 0.001**
Gender				0.486
Male [a]		0		
Female		-3.98	-15.26 to 7.30	0.486
Surgery Type				**< 0.001**
Laparoscopic cholecystectomy [a]		0		
Umbilical hernia repair		-23.64	-36.10 to -11.18	**< 0.001**
Ventral hernia repair		4.94	-8.47 to 18.35	0.467
Inguinal hernia repair		17.30	4.01 to 30.59	**0.011**
Post-VLCD fat mass		0.66	0.32 to 1.00	**< 0.001**

Statistically significant *p*-values < 0.05 are highlighted in bold.

Abbreviation: CI: Confidence interval.

Dependent variable: Surgical time.

[a] Reference category.
